# The impact of COVID-19 on national program of colorectal cancer screening in Tehran, Iran: a multicenter study

**DOI:** 10.1186/s12885-023-11111-x

**Published:** 2023-07-05

**Authors:** Amir Sadeghi, Hamid Asadzadeh Aghdaei, Mohammad Amin Khalafi, Ehsan Nazemalhosseini-Mojarad, Pardis Ketabi Moghadam, Mohammad-Reza Sohrabi

**Affiliations:** 1 Research Institute for Gastroenterology and Liver Diseases (RIGLD), Shahid Beheshti University of Medical Sciences (SBMU), Taleghani Hospital, Tehran, Iran; 2 Community Medicine Department, School of Medicine, Shahid Beheshti Medical University, Tehran, Iran

**Keywords:** Colonoscopy, Colorectal neoplasms, COVID-19, Early detection of cancer, Occult blood

## Abstract

**Background:**

The COVID-19 pandemic has affected all aspects of the healthcare system, including prevention, treatment, rehabilitation of diseases and health education; access to essential therapies; allocation of finance & facilities to health issues, and governance of diseases, including COVID-19 and other diseases. Consequently, the burden of COVID-19 was not only attributable to the multiorgan involvement and detailed presentation of the disease but also to the inadequate management of other diseases resulting from the exclusive allocation of resources and medical personnel to the pandemic crisis. Over the mentioned period, one observed deficiency was the lack of public and official favor for conventional screening protocols. To this end, this study aims to evaluate the impact of the COVID-19 pandemic on colorectal cancer (CRC) screening protocols at Shahid Beheshti University of Medical Sciences in Tehran, Iran, in an effort to identify individuals at risk for CRC and provide them with intensive screening and therapy.

**Methods:**

This is an observational study comparing the number of candidates for CRC screening referred to primary, secondary, and tertiary health-care centers under supervision of Shahid Beheshti University of Medical Sciences (SBMU), Tehran, Iran in a 2-year interval before and after COVID-19 pandemics. Patients with intermediate- and high-risk criteria for colorectal cancer were included in the study and were screened by fecal immunochemical test. Patients with positive or indeterminate fecal test results were further evaluated with colonoscopy in research institute for gastroenterology and liver diseases where is a tertiary referral center for CRC screening. Finally, the decrease percentage of screening tests and endoscopic findings during the pandemic period compared to pre-pandemic period was calculated and interpreted.

**Results:**

A significant decrease in the number of performed fecal immunochemical tests (FITs), referred positive FITs, and referred patients with positive alarm signs to the Research Institute of Gastroenterology and Liver Diseases (RIGLD) center inevitably led to a considerable decrease in the number of endoscopic findings, including high-risk adenomas, sessile serrated polyps, and even early-stage colorectal cancers (CRCs).

**Conclusion:**

The disruption of screening protocols caused by the COVID-19 pandemic appears to increase the number of patients with high-grade and end-stage CRCs referred in the near future.

**Supplementary Information:**

The online version contains supplementary material available at 10.1186/s12885-023-11111-x.

## Introduction

A global pandemic of COVID-19 was declared in March of 2020 [1–3]. Since then, the disease burden has been evaluated for different aspects of health issues [4–7]. Meanwhile, screening protocols are experiencing significant disruptions. They would benefit greatly from being reviewed by credible centers. The incidence of cancer screenings for breast cancer, CRC, and cervical cancer decreased significantly alongside the COVID-19 outbreak [5, 8–10]. Statistics show that we can expect a significant increase in these cancers’ incidence, mortality, and morbidity in the near future due to a lack of screening. In terms of CRC, there are reports corroborating a significant decrease (varying from 50%-80%) in the number of screening colonoscopies to prevent from spreading of COVID-19 to healthcare providers and patients [11–15]. According to the reports of the American College of Gastroenterology (ACG) [16], the American Gastrointestinal Association (AGA) [17], and the National Comprehensive Cancer Network (NCCN) [18], the role of effective screening protocols in the reduction of CRC is evident. Therefore, it is not surprising that the incidence of CRC in Iran will increase in the future. The national program of CRC screening was launched by SBMU in 2015. The RIGLD located in Taleghani Hospital, Tehran, Iran under supervision of SBMU was equipped with a multidisciplinary system including expert gastroenterologists, genetic consultants, CRC surgeons, and psychological consultants for CRC screening. 10 primary healthcare centers depicted in the Supplementary in city of Tehran and suburbs under the supervision of the RIGLD center, SBMU would evaluate the general population for CRC screening and would deliver FIT kits to the candidates of CRC screening. Eventually, they would determine eligible participants for further evaluations with colonoscopy. Thousands of participants aged 50–75 yearly with positive FITs, positive alarm signs, or positive first-degree relatives (FDRs) would send to the RIGLD center for colonoscopy screening. However, this strategy was greatly affected by the COVID-19 pandemic at the end of 2019. The present study aims to evaluate the percentage of public participation in the CRC screening program over a 2-year period before and after the initiation of the pandemic in northern and eastern Tehran. Such studies are valuable since a decrease in the number of CRC screenings mandates new strategies for the normalization of the burden of disease by precise diagnostic and therapeutic methods.

## Materials and methods

### The process of study (Target population, Questionnaire design, The need for intervention, Data collection)

**Fig. 1:**
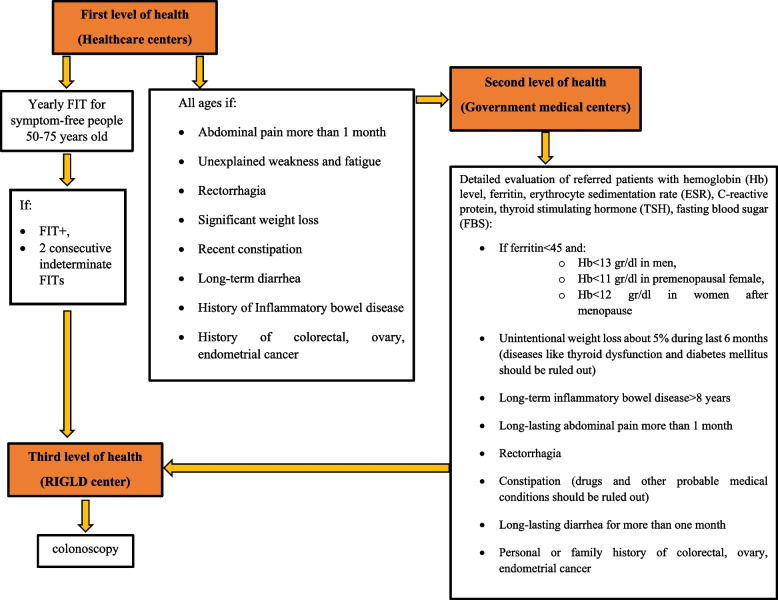
The process of referral to three levels of healthcare centers

The present study is an observational study on population referred to the healthcare centers for CRC screening in a 2-year interval before and after the COVID-19 pandemic under supervision of SBMU as depicted in the Supplementary. People were included in the study using total population sampling technique since the entire population referred to the mentioned healthcare centers were entered the study during the specified time period. Participants were stratified into 3 groups of low-risk, intermediate-risk, and high-risk for CRC as depicted in Table 1. Participants at low-risk for CRC were excluded from study. Then, the eligible participants were evaluated for the number of performed FITs, positive FITs, positive alarm signs, referred positive FITs and positive alarm signs to the RIGLD center, and several colonoscopy findings before and after the onset of the COVID-19 pandemic over a two-year period. Consequently, we divided the study into two-year intervals of pre-pandemic and pandemic years. The pre-pandemic phase started on January 1, 2018, and ended on December 1, 2019. The pandemic phase was calculated from December 1, 2019, to November 1, 2021. As reflected in Table 1, patients labeled as intermediate- and high-risk for CRC in the primary healthcare centers (supervised by SBMU) were included in the study and were stratified into two groups termed pre-pandemic and during-pandemic. The patient referral process to the RIGLD center is managed by three levels of healthcare centers, as depicted in Fig. 1. Registered nurses in the primary healthcare centers fill in the questionnaires concerning demographic characteristics, history of rectal bleeding, unexplained abdominal pain, recent constipation, weight loss, family or personal history of colorectal/stomach/ovary/uterus/renal cancer, and family or personal history of inflammatory bowel diseases (IBD) for all individuals referred to the centers at all ages after signing a written informed consent form. Participants aged 50–75 with negative answers are allocated to the intermediate-risk group for CRC. They are screened using FIT kits. Patients with positive or two consecutive indeterminate FIT tests are directly sent to the tertiary center, RIGLD, for further colonoscopy evaluations. People with positive answers in the first questionnaire are then referred to secondary healthcare centers, where they are visited by trained family physicians or general practitioners. The positive symptoms are assessed by complementary laboratory tests and detailed medical history. They are subsequently referred for further investigations with colonoscopy, upper GI endoscopy, and genetic consultation if required. Bowel preparation is thoroughly explained for candidates of colonoscopy by trained nurses in healthcare centers. Patients older than 60 and those with a history of diabetes, hypertension, or other cardiovascular diseases must undergo a cardiac consultation before the procedure. Table 1 also outlines the preferred CRC screening method for each group. The colonoscopy findings are documented in the RIGLD center. Further procedures in the RIGLD center include surgery, immunohistochemical tests (IHC), genetic consultation, and advanced endoscopic procedures based on the colonoscopy results. Participants without a complete colonoscopy, as defined by cecal or terminal ileum intubation or inadequate preparation (Boston Bowel Preparation Score [BBPS] of less than 2 in each segment of the colon), are encouraged to undergo a second colonoscopy. Notably, participants with incomplete colonoscopies were excluded from the present study.

**Table 1:** Risk stratification of patients for CRC

**Risk stratification**	**Included participants**	**Age of onset & intervals**^**a**^** for CRC screening**	**Method of choice for CRC screening**
**Low-risk group**	People under 50 years old without alarm signs, positive family history for colorectal cancer, positive family history for other genetic syndromes related to colorectal cancer, personal history of colorectal cancer, and personal history of colorectal polyps	No need for CRC screening	No need for CRC screening
**Intermediate-risk group**	People 50–75 years old without alarm signs, positive family history for colorectal cancer, positive family history for other genetic syndromes related to colorectal cancer, personal history of colorectal cancer, and personal history of colorectal polyps	50–75 years^b^	FIT
**High-risk group**	Patients with positive family history for genetic syndromes related to colorectal cancer	Based on genetic syndrome	Colonoscopy
	Positive family history for advanced adenoma/colorectal cancer in one first degree relative less than 60 years old or ≥ 2 first degree relatives at any age	At 40 years or 10 years prior to the youngest age at which a person is involved and then every 5 years	
	Positive family history for colorectal cancer or advanced adenoma in one first degree relatives at age ≥ 60 years old	At age 40 and then every 10 years	
	Positive personal history for inflammatory bowel diseases	8 years from onset of disease	
	Positive personal history for colorectal cancer	At years 1 and 3 after surgery then every 5 years	
	Positive personal history for advanced adenomas/high-risk sessile serrated polyps/hyperplastic polyps	3 years later	
	Positive personal history for low-risk adenomas/sessile serrated polyps/hyperplastic polyps	7–10 years later for adenomas, 5–10 years later for sessile serrated polyps, 10 years later for hyperplastic polyps	
	Positive personal history for more than 10 adenomas/20 hyperplastic polyps	1 year later	
	Positive personal history of rectal bleeding accompanied by age ≥ 50 years old or abdominal pain, change in bowel habit, weight loss, anemia of iron deficiency	Immediately	
	Positive personal history for unexplained abdominal pain/weight loss/anemia of iron deficiency/change in bowel habit	Immediately	
	Positive personal history for abdominal or rectal mass	Immediately	

^a^Intervals are considered by default when the result of colonoscopy is unremarkable. Intervals for next colonoscopies are decided individually when the result is abnormal

^b^Decision-making for colorectal cancer screening in participants older than 75 years old was individualized based on performance and comorbidities

### Statistical analysis

Categorical variables are expressed as numbers and percentages. χ2 or Fisher’s exact test, where appropriate, was used for analysis of categorical variables. Continuous variables are expressed as medians, or as means and standard deviation, and 95%CI as appropriate. All analyses were performed using SPSS version 21.0 (SPSS INC, Chicago, IL, United States). A two-tailed *P* < 0.05 was considered statistically significant. The decrease in the number of screening tests and findings of colonoscopy during the pandemic period in comparison with pre-pandemic period was computed as percentage decrease = (N during pandemic − N pre-pandemic)/N pre-pandemic. The reduction percentage in the number of people screened and in the number of endoscopic findings were considered as primary and secondary endpoints of the study, respectively.

### Ethical considerations

Ethical approval for this study was provided by RIGLD, SBMU before data collection and data analysis regarding the approval of world health organization (WHO) and all gastroenterology societies for mass screening of CRC using FIT kits and/or colonoscopy evaluations.

## Results

**Fig. 2:**
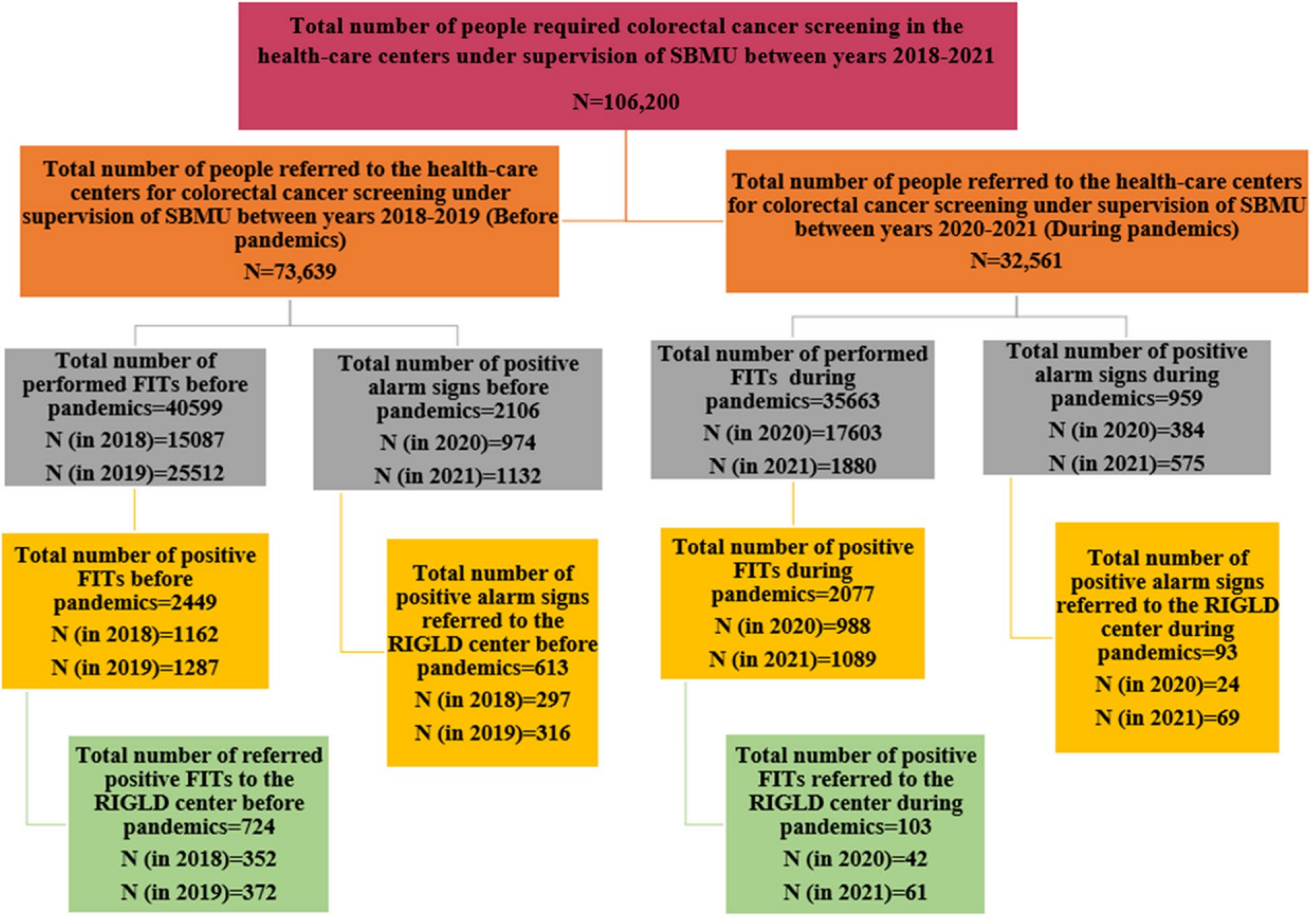
Patients’ contributions in comprehensive colorectal cancer screening during 2018–2021

**Fig. 3:**
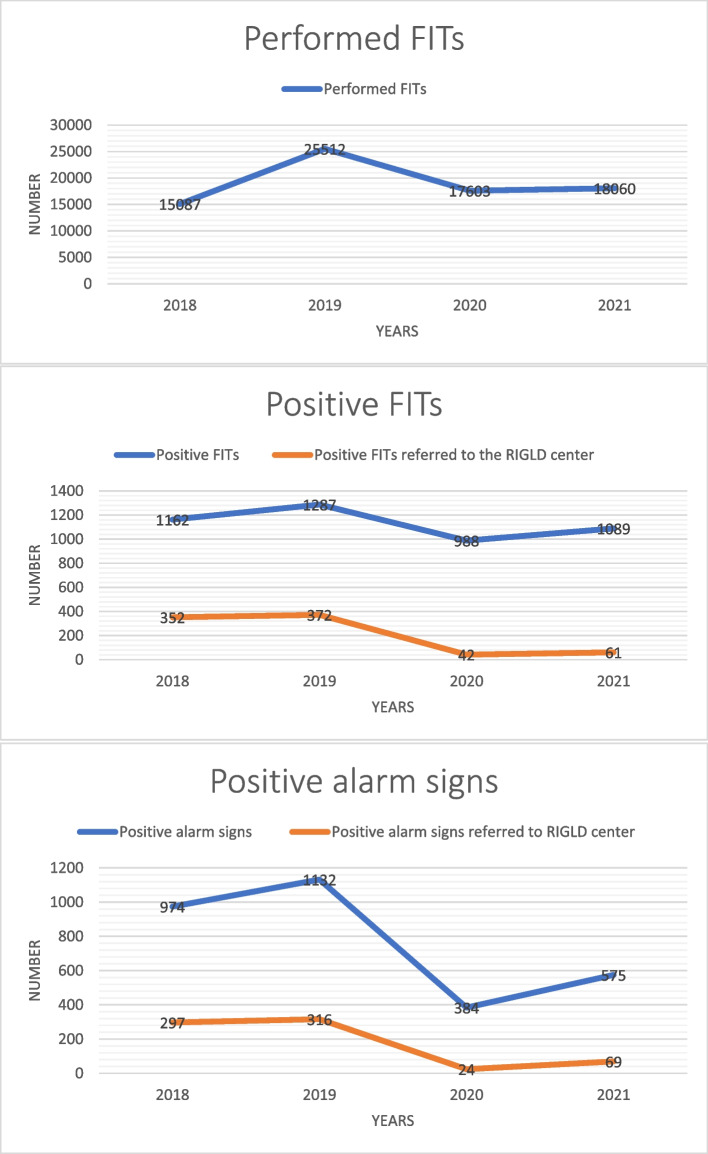
The first plot shows the number of performed FITs in healthcare centers under supervision of SBMU over a 2-year period before and after the onset of the COVID-19 pandemic. As could be driven from the first plot, the total number of performed FITs has decreased by 12.16% over the first 2 years after the onset of the pandemic in contrast to the 2 years before the pandemic. The second plot shows the number of individuals with positive alarm signs referred to healthcare centers under supervision of SBMU and the RIGLD center during 2 years before and after the onset of the COVID-19 pandemic. The total number of patients with warning signs referred to the healthcare centers has decreased by 54.46% over the first 2 years after the onset of the pandemic in contrast to the 2 years before the onset of the pandemic. This decrease is 84.83% for patients with alarm signs referred to the RIGLD center for colonoscopy. As it is reflected on the third plot, the number of positive FITs in the healthcare centers during a 2-year interval after the onset of the COVID-19 pandemic has decreased by 15.19% in contrast to the 2-year interval before the pandemic. This decrease is 85.77% for positive FITs referred to the RIGLD center for colonoscopy which is significantly higher than the percentage of the decrease is seen for patients with positive FIT in the healthcare centers. The rational for this difference is the possibility of at home FITs kits leading to a weaker effect of the COVID-19 on the number of positive FITs compared to the positive FITs referred to the RIGLD center for colonoscopy

The number of patients contributing to the national CRC screening program is depicted in Fig. 2; the total number of patients who required CRC screening based on their age and symptoms was estimated to be approximately 106,200 from January 1, 2018, to November 1, 2021. Among them, 73,639 patients were referred to healthcare centers before the COVID-19 pandemic, and 32,561 patients were referred during the pandemic. In total, 76262 FITs were performed; 40599 FITs were performed before the pandemic, and the remainder during the pandemic. This shows a significant decrease (*P*-value < 0.05) in performed FITs after pandemic in comparison with pre-pandemic period. A detailed number of performed FITs is shown in Fig. 2. The total number of patients advised to undergo colonoscopy due to positive alarm signs was 3065. Among them, 2106 were allocated to the pre-pandemic group, and 959 were allocated to the during-pandemic group. It suggests that COVID-19 pandemic has been ended up a significant decrease in the number of referred patients with positive alarm signs to the primary healthcare centers (*P*-value = 0.00001). Figure 2 shows the detailed number of patients with positive alarm signs identified in healthcare centers and referred to the RIGLD center for screening colonoscopy before and after the pandemic. The total number of positive FITs was 2449 and 2077 before and during the pandemic, respectively. Before the pandemic, 613 patients with positive alarm signs were ultimately referred to the RIGLD center for colonoscopy. During the pandemic, this number decreased to 93. The total number of patients with positive FIT accepted for colonoscopy at the RIGLD colonoscopy center before and during the pandemic was 724 and 103, respectively. The detailed number of patients with positive FITs detected in the healthcare centers and referred to the RIGLD center for screening colonoscopy before and after the pandemic is shown in Fig. 2. The first plot of the Fig. 1 shows the number of performed FITs in healthcare centers under supervision of SBMU over a 2-year period before and after the onset of the COVID-19 pandemic. As could be driven from the first plot in the Fig. 3, the total number of performed FITs has decreased by 12.16% over the first 2 years after the onset of the pandemic in contrast to the 2 years before the pandemic. The second plot in the Fig. 3 shows the number of individuals with positive alarm signs referred to healthcare centers under supervision of SBMU and the RIGLD center during 2 years before and after the onset of the COVID-19 pandemic. The total number of patients with warning signs referred to the healthcare centers has decreased by 54.46% over the first 2 years after the onset of the pandemic in contrast to the 2 years before the onset of the pandemic. This decrease is 84.83% for patients with alarm signs referred to the RIGLD center for colonoscopy. As it is reflected on the third plot in the Fig. 3, the number of positive FITs in the healthcare centers during a 2-year interval after the onset of the COVID-19 pandemic has decreased by 15.19% in contrast to the 2-year interval before the pandemic. This decrease is 85.77% for positive FITs referred to the RIGLD center for colonoscopy which is significantly higher than the percentage of the decrease is seen for patients with positive FIT in the healthcare centers. The rational for this difference is the possibility of at home FITs kits leading to a weaker effect of the COVID-19 on the number of positive FITs compared to the positive FITs referred to the RIGLD center for colonoscopy. The detailed number of performed FITs, positive FITs and positive FITs referred to the RIGLD center for colonoscopy, individuals with warning signs and individuals with alarm signs referred to the RIGLD center for colonoscopy as well as number of patients with endoscopic findings in the 2-year intervals before and after the onset of the COVID-19 is seen in Table-2. Accordingly, the number of performed FITs, positive FITs, patients with positive alarm signs referred to the primary healthcare centers was decreased by 12.16%, 15.19%, 54.46% from 40599, 2449, 2106 to 35663, 2077, 959, respectively. Between January 1, 2018, and December 1, 2019, 1,337 individuals were referred to the RIGLD clinic due to positive FIT or positive alarm signs. In the two years following the pandemic’s start, from December 1, 2019, to November 1, 2021, this number decreased by 85% to 196. From 2018 to 2019, 724 and 613 of these individuals were found to have positive FIT and warning signs, respectively. Between 2019 and 2021, this number fell by 86% to 103 for positive FIT and by 84083% to 93 for alarm signs. (*P*-value < 0.05). As reflected on Table-2, a significant reduction in the percentage of patients with warning signs and positive FITs referred to the RIGLD center for colonoscopy is detected after the onset of the pandemic in contrast to the pre-pandemic period (*P*-value < 0.05). The estimated number of patients with positive alarm signs was 382 for positive family history in first-degree relatives, 23 for iron deficiency anemia, 90 for rectorrhagia, 53 for a change in bowel habit, 48 for abdominal pain, and 9 for weight loss before the pandemic. After the pandemic, there was a significant decrease of approximately 87% for participants with positive first-degree relatives, 96% for iron deficiency anemia, 91% for rectorrhagia, 81% for a change in bowel habit, 52% for abdominal pain, and 89% for weight loss compared to the period before the pandemic. Along with the significant decrease in participants referred to the RIGLD center, the number of endoscopic findings also decreased significantly (*P*-value < 0.05). Consequently, the number of polyps, CRCs, and IBDs decreased from 439, 26, and 31 before the pandemic to 65, 5, and 16 after the onset of the pandemic, which corresponds to a decrease of approximately 85%, 81%, and 48%, respectively. These changes are summarized in Table 2. There is a major limitation in this study that could be addressed in the next studies. The focus of this study was the number and percentage of candidates for screening colonoscopy before and after the onset of the COVID-19. Furthermore, the number and percentage of patients with abnormal endoscopic findings were compared before and after the pandemic. However, detailed number, characteristics, and severity of endoscopic findings as a mine of information were not discussed in details in the study. To assume the slope angle of the increasing number of CRCs, advanced CRCs, and advanced polyps in the future due to the destructive effect of the COVID-19 on screening protocols, we should have mentioned the detailed number of high-risk adenomas and CRCs. Consequently, it requires more detailed studies in this field. Additionally, as with the majority of studies conducted in a particular geographic location, the results must be interpreted and extrapolated to other nations with caution due to various healthcare facility barriers in different cultures.

**Table 2:** Patients referred to the health-care centers and RIGLD center for colorectal cancer screening

**Date**	**Referral to health-care centers**			**Referral to the RIGLD**^d^** center**										**Patient’s contribution percentage for colonoscopy in case of FIT + (%) in RIGLD**	**Patient’s contribution percentage for colonoscopy in case of positive alarm signs (%) in RIGLD**	**Endoscopic findings in the RIGLD center**			
	**Total FITs**^a^ (n)	**FIT + (n)**	**Alarm signs + (n)**	**FIT + (n)**	**Positive alarm signs**								**Sum (n)**			**Polyps (n)**	**CRC**^e^ (n)	**IBD**^f^ (n)	**Nl (n)**
					**FH**^b^** + (n)**	**IDA**^c ^(n)	**Rectal bleed (n)**	**Bowel habit change (n)**	**Abd. pain (n)**	**Weight loss (n)**	**Other (n)**	**Sum (n)**							
**2018–2019**	40599	2449	2106	724	382	23	90	53	48	9	8	613	1373	29.563%	29.107%	439	26	31	841
**2020–2021**	35663	2077	959	103	50	1	8	10	23	1	0	93	196	4.959%	9.697%	65	5	16	110
**Decrease percentage during pandemics (%)**	-12.16%	-15.19%	-54.46%	-85.77%	-87%	-96%	-91%	-81%	-52%	-89%	-100%	84.83%	-85%	-83.23%	-66.68%	-85%	-81%	-48%	-87%
***P*-value**	0.00001	0.00001	0.00001	0.00001										0.00001	0.00003	0.00001			

Table 2 The detailed number of performed FITs, positive FITs and positive FITs referred to the RIGLD center for colonoscopy, individuals with warning signs and individuals with alarm signs referred to the RIGLD center for colonoscopy as well as number of patients with endoscopic findings in the 2-year intervals before and after the onset of the COVID-19. For analysis of categorical variables X^2^ was used and if needed Fisher’s exact test was used

^a^*FIT* Fecal Immunochemical Test

^b^*FH* Family History

^c^*IDA* Iron Deficient Anemia

^d^*RIGLD* Research Institute for Gastroenterology and Liver Diseases

^e^*CRC* Colorectal Cancer

^f^*IBD* Inflammatory Bowel Disease

## Discussion

It is safe to assume that the burden of the COVID-19 pandemic on society is not only limited to the complicated consequences of the infection directly caused by the virus [19] but also due to the shortage of facilities and time management in other aspects of the health system in response to the extent of pandemic requirements [5, 6]. One of the irreparable consequences of the pandemic is the disruption of global cancer screening protocols [20]. Consequently, we would anticipate a sudden increase in the number of patients presenting with advanced stages of cancer in the near future if we were unaware of the issue and failed to address the challenge. According to this study, the number of individuals referred to the RIGLD center for screening colonoscopy during the COVID-19 pandemic has decreased significantly. Similarly, the number of endoscopic findings, including various polyps, CRCs, and IBDs, also decreased. These statistics can predict the number of patients who might be referred with advanced-stage CRC in the future. Consequently, the survival rate and life expectancy of patients with CRC are significantly reduced. In this regard, reports from global healthcare systems indicate that the absence of CRC screening protocols would have irreversible consequences in some areas of the world. After the onset of the pandemic, D’Ovidio et al. in Italy found that the acceptance rate of patients with FIT + for colonoscopy decreased by approximately fourfold. A follow-up of this group of patients revealed an eightfold increase in CRCs and a 1.9-fold increase in high-risk adenomas [21]. These findings were consistent with a survey conducted in Vives, Spain, which showed a decrease of about 5.1% and 8.9% in participation and adherence to a screening colonoscopy, respectively, from January to March 2020 [22] and the study of London et al., reporting a decrease of 5.6% in February 2020, 39.4% and 84.5% in March and April 2020, respectively [23]. A comprehensive review of databases by Mazidimoradi et al. reported similar results. Total colonoscopies showed a decrease of 65.7%, surveillance colonoscopies showed a decrease of 44.6 to 79%, and referrals to colonoscopy units represented a decrease of about 43% [24]. This was also demonstrated by Ricciardiello et al., who reported a significant increase in advanced CRC from 26 to 29% and even 33% after 0–3, 7–12, and > 12 months of delay in CRC screening due to the COVID-19 virus. Along with the advanced stages of CRC, the number of cancer-related deaths also increased [25]. These statistics align with the study of Roshandel et al., who expects a 5-time increase in the number of CRCs in Iran by 2025 [26, 27]. In contrast, Jidkova et al. found that invitation coverage, patients’ willingness to be screened, and screening intervals were not affected in the colorectal, cervical, and breast cancer screening programs following the conclusion of the first months of the COVID-19 pandemic [28]. Consequently, additional research is required in this field to definitively determine the impact of the COVID-19 pandemic on cancer screening programs in various parts of the world with different protocols. Healthcare centers should be organized and equipped with mass screening facilities in areas where a lack of screening is felt to solve the problem in areas like the one used in the current study. Such research helps to compensate for the lack of cancer screening during the pandemic. Many studies focus on new ways to improve the life expectancy of people utilizing organized expansion of screening protocols. These studies emphasize that mass screening is not only limited to the patients referred to the clinics but also people at home [11–13]. SBMU oversaw establishing such programs in healthcare centers by dispatching health ambassadors to the suburbs and rural areas to provide the necessary education and facilities for at-home FIT kits. Nonetheless, proportional improvements in telehealth, recruitment, and personnel training that inform the general population, healthcare center networking, and tertiary center governance for advanced screening tools are anticipated. Shreya et al. explain that organized outreach is more effective than provider-initiated screening. The results show an increase from approximately 40% to over 80% in total CRC screenings employing organized outreach in large healthcare systems [2, 4]. They have introduced telehealth as the method of choice for improving screening protocols. Telehealth allows centers to send FIT kits to patients’ homes, collect them, and monitor their results. This would increase the number of screening program participants. Other introduced methods to compensate for CRC screening deficiencies due to COVID-19 are automated patient messaging systems, improved tracking reminder systems, patient risk assessment and tailoring patient education, and gastroenterologists’ commitment to expanding open-access colonoscopies. Future studies can help evaluate the effectiveness of these methods [29–31]. In this method, patients at high risk for CRC will undergo a colonoscopy and be followed for longer.

Accordingly, the study results indicate that the number of FITs performed during the pandemic has decreased by approximately 12.157% compared to before the pandemic. This decrease, while statistically significant (*P*-value < 0.05), is less than the reduction percentage of positive FITs and positive alarm signs referred to the RIGLD center and subsequent endoscopic findings in the RIGLD center. This is likely because, despite restrictions attributed to COVID-19, the mailing of FIT kits has continued. Due to the pandemic, however, a significant decline in the number of patients referred to the RIGLD center for positive FITs (-86%) or positive alarm signs (-85%) was inevitable. The percentage of patients with positive FITs and positive alarm signs decreased from 29.563% and 29.107% to 4.959% and 9.697%, respectively, as shown in Table 2. These statistics indicate that the referral rate of patients from healthcare facilities to the RIGLD center has decreased by 83.22% for positive FITs and by 66.68% for positive alarm signs. Even though the contribution percentage of patients with positive FITs and positive alarm signs has decreased significantly (*P*-value < 0.05) due to the establishment of COVID-19, the final results suggest that symptomatic patients with positive FITs were referred for screening colonoscopy more frequently than asymptomatic patients during the first two years of the pandemic. The percentage of referrals to the RIGLD center before the pandemic, which is approximately 29 percent for positive FITs and positive alarm signs, suggests that a significant number of patients with positive alarm signs and positive FITs have missed follow-up colonoscopies. Screening protocols must be revised to convince participants to accept FIT as a two-step screening test requiring further colonoscopic evaluation if the results are positive. If they have positive CRC warning signs, they should also be persuaded to undergo a colonoscopy as a first step. As a result, the present study indicates that efforts should be made to eliminate the limitations mentioned regarding CRC screening. There is a major limitation in this study that could be addressed in the next studies. The focus of this study was the number and percentage of candidates for screening colonoscopy before and after the onset of the COVID-19. Furthermore, the number and percentage of patients with abnormal endoscopic findings were compared before and after the pandemic. However, detailed number, characteristics, and severity of endoscopic findings as a mine of information were not discussed in details in the study. To assume the slope angle of the increasing number of CRCs, advanced CRCs, and advanced polyps in the future due to the destructive effect of the COVID-19 on screening protocols, we should have mentioned the detailed number of high-risk adenomas and CRCs. Consequently, it requires more detailed studies in this field. Additionally, as with the majority of studies conducted in a particular geographic location, the results must be interpreted and extrapolated to other nations with caution due to various healthcare facility barriers in different cultures. Entry of the majority of population covered by 10 major healthcare centers under supervision of SBMU leading to the recruitment of a large number of individuals for a large sample size is the strength of the study.

## Conclusion

Disruption of screening protocols due to the COVID-19 pandemic will increase the number of patients referred with high-grade and end-stage CRCs in the near future. This requires healthcare providers to act. As stated in the study, a reduction in the number of performed FITs, referred positive FITs, and referred patients with positive alarm signs to the RIGLD center inevitably led to a reduction in the number of endoscopic findings, including high-risk adenomas, sessile serrated polyps, and even early-stage CRCs. Based on the data, there is an urgent need to strengthen resources to promote CRC screening.

## Supplementary Information

**Additional file 1: Supplemetary Material.** The map of Tehran province is depicted with black color and covered health-care centers under supervision of Shahid Beheshti University of Medical Sciences are presented with different colors.

## Data Availability

The datasets used and/or analyzed during the current study available from the corresponding author on reasonable request.

## Abbreviations

ACG: American College of Gastroenterology
AGA: American Gastrointestinal Association
BBPS: Boston Bowel Preparation Scale
CRC: Colorectal Cancer
FDR: First degree relatives
FIT: Fecal immunochemical test
IBD: Inflammatory Bowel Disease
IHC: Immunohistochemistry
NCCN: National Comprehensive Cancer Network
RIGLD: Research Institute for Gastroenterology and Liver Diseases
SBMU: Shahid Beheshti University of Medical Sciences
