# Supplementary material for: The impact of COVID-19 on national program of colorectal cancer screening in Tehran, Iran: a multicenter study
Source: BMC Cancer. 2023 Jul 5;23:627. doi: 10.1186/s12885-023-11111-x (PMC10320866; doi:10.1186/s12885-023-11111-x)

Supplemetary Material The map of Tehran province is depicted with black color and covered health-care centers under supervision of Shahid Beheshti University of Medical Sciences are presented with different colors

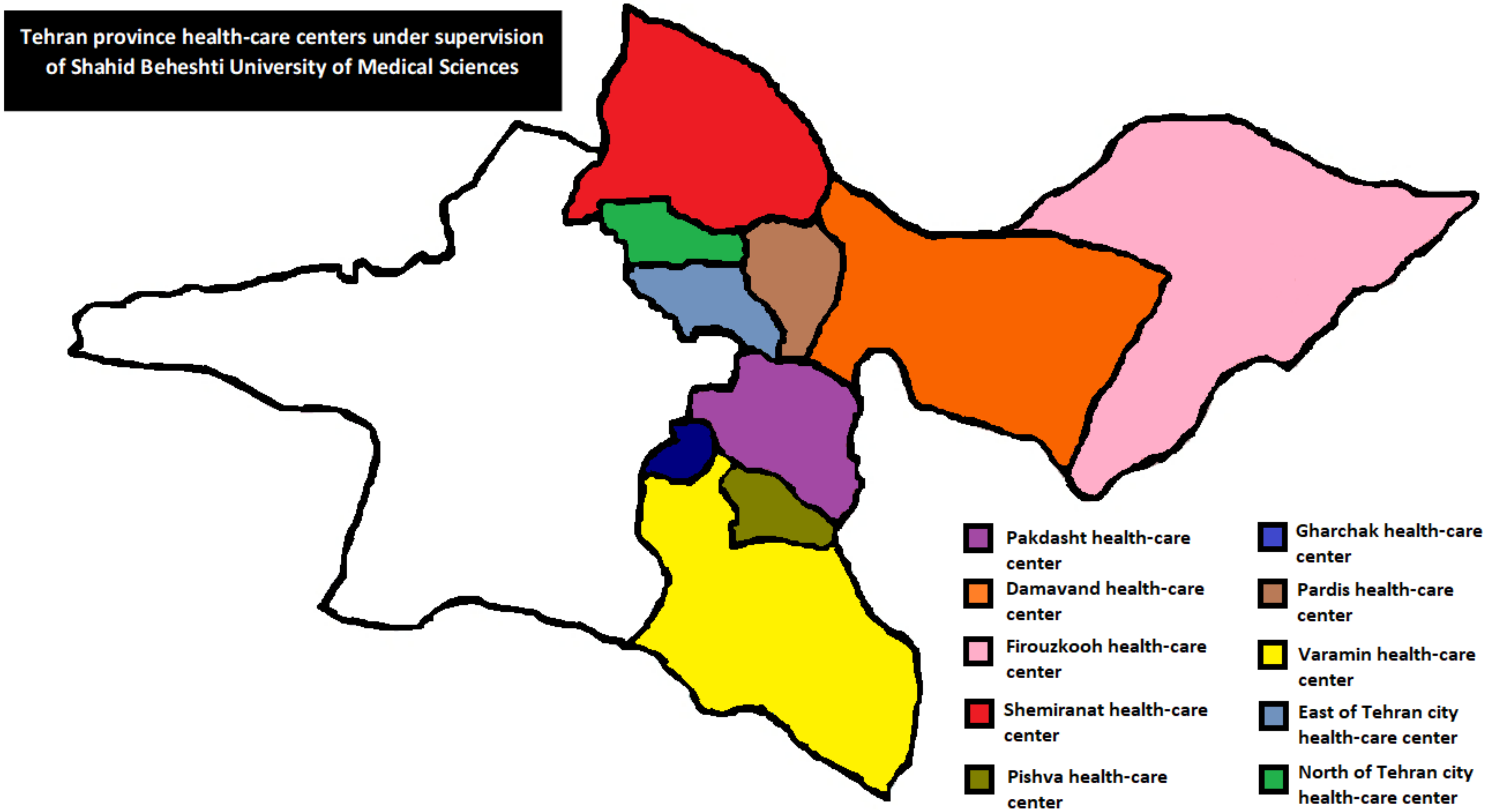

Supplement: Supplementary file 1 — Additional file 1: Supplemetary Material. The map of Tehran province is depicted with black color and covered health-care centers under supervision of Shahid Beheshti University of Medical Sciences are presented with different colors. [file 12885_2023_11111_MOESM1_ESM.pdf]
